# Management of Proximal Humerus Fracture With Concurrent Axillary Artery Injury Using a Saphenous Vein Graft and Reverse Shoulder Prosthesis: A Case Report

**DOI:** 10.7759/cureus.69211

**Published:** 2024-09-11

**Authors:** Adnane Lachkar, Najib Abdeljaouad, Hicham Yacoubi

**Affiliations:** 1 Faculty of Medicine and Pharmacy of Oujda, Mohammed First University, Oujda, MAR; 2 Department of Orthopedic Trauma, Mohammed VI University Hospital, Oujda, MAR; 3 Department of Orthopedics, Mohammed VI University Hospital, Oujda, MAR

**Keywords:** axillary artery, complex proximal humerus fracture, great saphenous vein grafting, revascularization, reverse shoulder arthroplasty

## Abstract

This case report details the management of a 60-year-old male who sustained a complex proximal humerus fracture with an axillary artery injury following a fall. Initial assessment revealed a fractured humeral head and complete occlusion of the axillary artery, which was repaired using a saphenous vein graft. Subsequently, the patient underwent reverse shoulder arthroplasty (RTSA) after the limb stabilized. The staged approach, prioritizing urgent vascular repair followed by delayed orthopedic intervention, proved effective. The patient achieved satisfactory functional recovery with an improved range of motion and no pain. This case highlights the importance of prompt diagnosis and treatment of vascular injuries in complex proximal humeral fractures and supports the use of saphenous vein grafting and RTSA as effective management strategies.

## Introduction

Proximal humerus fractures make up about 5% of all fractures involving long bones [1]. Injuries to arteries occurring alongside these fractures are quite uncommon [2,3]. Vascular injuries in the upper limbs are more often the result of penetrating trauma, with axillary artery damage due to falls or road accidents being particularly rare. This infrequency may be due to the axilla's ample loose connective tissue and the soft tissue space surrounding it, which might lower the risk of vascular injury due to the lack of tight anatomical compartments.

Restoration of the axillary artery can be accomplished either through direct repair or by utilizing grafts, such as those made from the saphenous vein [4]. Complex fractures of the proximal humerus, including three-part, four-part, or head-splitting types, pose considerable treatment challenges. Non-surgical management, open reduction with internal fixation, or hemiarthroplasty often lead to suboptimal and unpredictable results [5]. For older patients with such complex fractures, reverse shoulder arthroplasty has become the treatment of choice, offering better functional recovery, improved range of motion, and higher patient satisfaction compared to alternative surgical methods [5,6].

In this report, we present a case of successful reconstruction of the axillary artery using a saphenous vein graft, combined with reverse shoulder prosthesis implantation, in a patient with a complex proximal humerus fracture, including a head-splitting component.

## Case presentation

**Figure 1:**
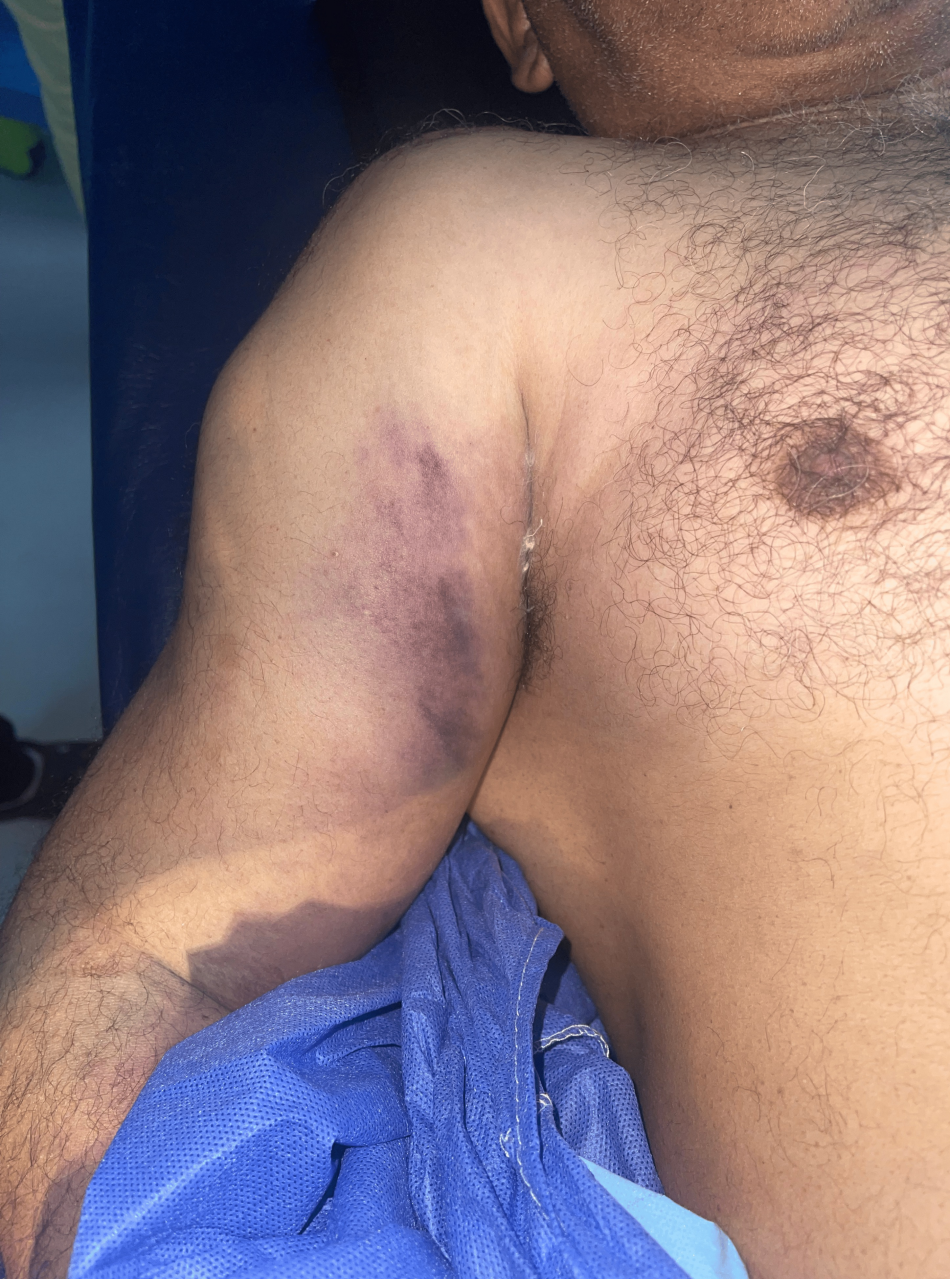
Clinical findings of the right shoulder, including deformity and associated bruising.

A 60-year-old male patient presented to the emergency department following a fall from a height of approximately 2.5 meters from a staircase. His medical history included chronic alcohol consumption and a 40-year history of smoking. The patient fell onto his right shoulder and immediately experienced severe pain. On physical examination, the right shoulder was found to be tender and swollen and exhibited restricted movement due to pain (Figure 1). Importantly, the clinical assessment revealed the absence of palpable pulses in the right upper extremity, specifically in the brachial, radial, and ulnar arteries.

**Figure 2:**
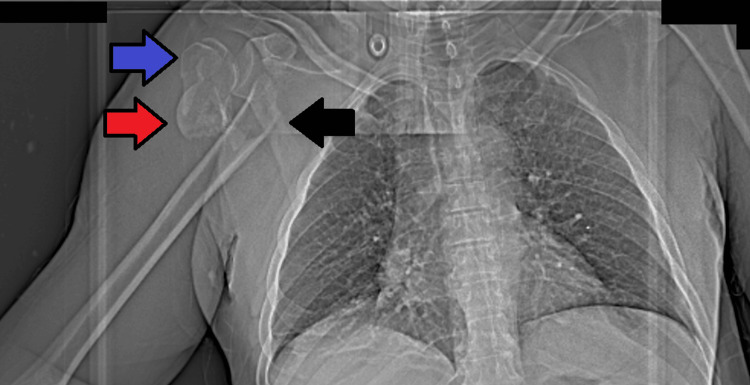
Anteroposterior scout view of chest CT showing a complex fracture of the right proximal humerus. The blue and red arrows indicate the splitted humeral head and the black arrow highlights the displaced proximal humeral shaft in the axilla.

**Figure 3:**
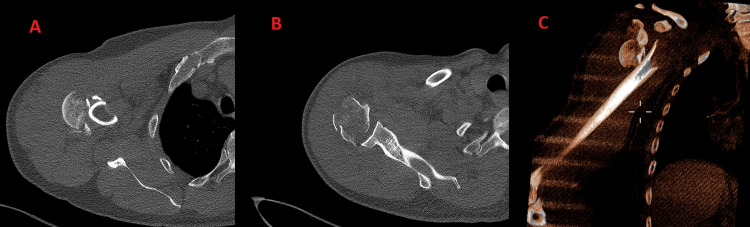
CT scan views of the right shoulder. A: The first fragment of the split humeral head. B: The second fragment of the split humeral head, positioned lateral to the glenoid fossa. C: Volume rendering view (VR view) from the CT scan of the right shoulder, demonstrating the comminution of the fracture.

Initial plain radiographs identified a fracture of the proximal humerus. Further evaluation with computed tomography (CT) revealed a complex proximal humeral fracture, including a split in the humeral head (Figures 2, 3). CT angiography showed a complete occlusion of the right axillary artery caused by the sharp edge of the fractured bone. Despite the absence of detectable pulses, capillary refill was present, and collateral circulation provided distal filling of the brachial artery.

**Figure 4:**
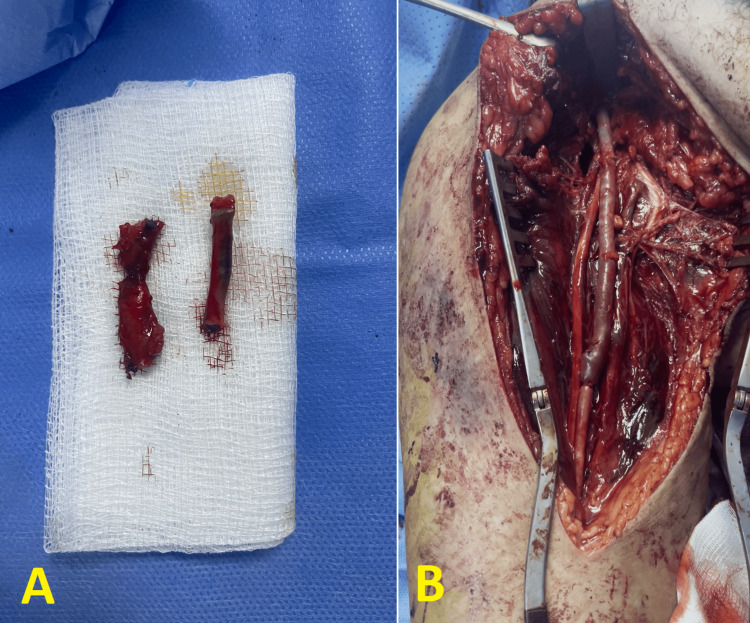
Perioperative view of the damaged axillary artery and the great saphenous vein graft used for repair. A: The excised damaged segment of the axillary artery illustrating the extent of the injury. B: The great saphenous vein graft after vascular continuity was restored through end-to-end anastomosis.

The patient was swiftly transferred to the operating room for emergency arterial intervention. An axillary incision was selected to expose the axillary artery for exploration. During surgery, a contused section of the distal axillary artery was identified, characterized by the absence of pulsations and lack of distal blood flow. Prior to further vascular procedures, temporary stabilization of the shoulder joint was achieved using wires. After achieving proximal and distal vascular control, the injured segment of the axillary artery was excised. The excised segment revealed an intimal flap that had occluded the arterial lumen. A 12-cm section of the proximal great saphenous vein was harvested from the ipsilateral thigh to serve as an interposition graft. Vascular continuity was re-established through end-to-end anastomosis using 7/0 sutures, which successfully restored pulsatile blood flow to the brachial, radial, and ulnar arteries on the affected side (Figure 4). The postoperative course was uneventful, and the limb remained viable.

**Figure 5:**
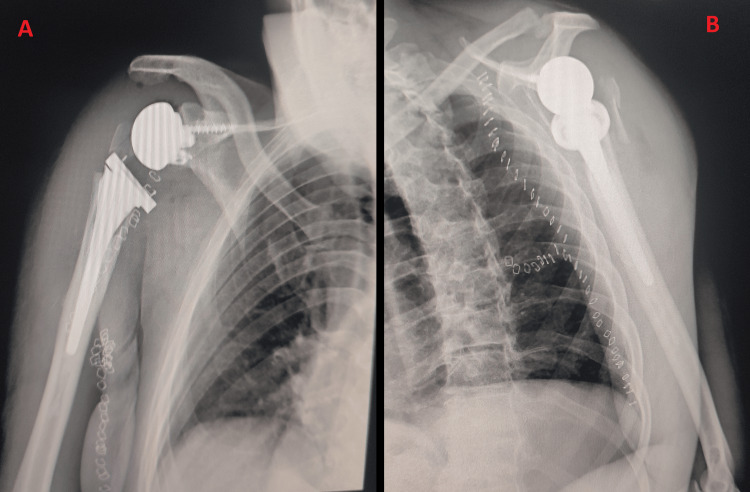
X-ray following reverse total shoulder arthroplasty (RTSA) implantation. A: Anteroposterior view. B: Lateral view.

Two weeks after the initial surgery, once the wound had healed and the shoulder swelling and inflammation had subsided, the patient underwent a secondary procedure to manage the humeral fracture using reverse total shoulder arthroplasty. The patient was placed in the beach chair position, and preoperative antibiotics along with tranexamic acid were administered according to the patient’s weight. A standard deltopectoral approach was employed. The previously placed K-wires were removed, and the axillary nerve was identified and carefully protected. The humeral head was excised, and the area was cleared of any bony fragments and articular debris. The glenoid was then exposed, prepared, and reamed. The glenosphere was implanted, followed by the preparation of the humerus and insertion of the prosthetic stem. The tuberosities were reattached thereafter (Figure 5). The entire operation lasted one hour and 45 minutes, with an estimated blood loss of 200 mL. The procedure was completed without any perioperative complications.

The patient was discharged to a rehabilitation facility one week after hospital admission. Postoperative management included a restriction on weight-bearing for three weeks, with physical therapy commencing thereafter. Internal rotation and abduction were avoided for the first 10 weeks following surgery. By five months postoperatively, the patient demonstrated satisfactory functional recovery, including 30 degrees of external rotation, 80 degrees of abduction, and 120 degrees of active forward flexion, all achieved without any associated pain.

## Discussion

Fracture-dislocations of the proximal humerus infrequently involve injuries to the axillary artery. Consequently, first responders may not routinely consider this possibility, which can lead to delays in diagnosis [7]. Additionally, the robust collateral circulation to the upper limb often results in subtle signs of ischemia. Rather than presenting with overt ischemic symptoms, patients may exhibit more subtle signs, including delayed capillary refill, reduced pulse volume, and lower pulse oximetry readings [8]. This underscores the significance of our case report in highlighting the necessity of a thorough vascular assessment, including pulse evaluation, in cases of complex proximal humeral fractures. Prompt intervention is critical to prevent severe hemorrhage or potential limb amputation resulting from axillary artery injuries. The presence of concomitant brachial plexus injuries can further complicate management and require careful assessment; however, in this instance, the brachial plexus was found to be intact. When arterial injury is clinically suspected, confirmation and localization are achieved through arteriography or CT angiography. Interposition grafting using the great saphenous vein is commonly accepted as the preferred technique for revascularization in these scenarios, due to its optimal size, ease of procurement, and compatibility with grafting and suturing [9,10].

In recent years, reverse total shoulder arthroplasty (RTSA), initially developed for managing massive, irreparable rotator cuff tears in elderly patients with or without glenohumeral arthritis, has increasingly been applied to trauma cases. RTSA is now considered by many surgeons as the most effective approach for complex proximal humeral fractures, especially in cases where previous surgical interventions have been unsuccessful [11,12]. For elderly patients with complex fractures involving a split humeral head, RTSA is often preferred [13]. It yields favorable functional outcomes, particularly when fractures are too complex for open reduction and internal fixation and where there is a substantial risk of avascular necrosis of the humeral head [12]. In this case, the timing of RTSA implantation was a critical consideration. Performing RTSA concurrently with venous grafting for the axillary artery injury was deemed impractical due to the urgent need for upper limb revascularization, the use of the axillary approach, and the potential instability of the patient resulting from hemorrhage or limb ischemia. Therefore, RTSA implantation was deferred until a few days after the initial procedure, allowing for stabilization and transition through the critical emergency phase. This strategy enabled a potentially simpler subsequent procedure, resulting in favorable functional outcomes following this severe and rare osteovascular injury of the upper limb.

## Conclusions

In this case, the management of a complex proximal humerus fracture with concurrent axillary artery injury demonstrated the effectiveness of a staged approach combining saphenous vein grafting and RTSA. The initial urgent intervention focused on revascularization using a saphenous vein graft to restore arterial continuity, which was essential for limb viability. Following stabilization and resolution of the acute phase, RTSA was successfully performed to address the complex fracture. This staged strategy allowed for optimal patient preparation and contributed to favorable functional outcomes. Our case underscores the importance of timely diagnosis and appropriate management in complex traumatic injuries involving both vascular and orthopedic components.
